# Assessing Pediatric Inter-Hospital Transfer: A single-center, Retrospective, Observational Study of Saudi Arabia’s National Life-Saving Protocol

**DOI:** 10.26502/jppch.74050130

**Published:** 2022-08-28

**Authors:** Hakem Alomani, Ahmed Ramadan, Gehad Omran, Mohamed Elbiomy, Mahmoud Elzonfly, Asma Alenazi, Njood AlBarrak, Ali A. Alakhfash, Ramesh K. Vishwakarma, Fawaz Alanzi, Yousef Alotaibi

**Affiliations:** 1 Consultant, Pediatric critical care, Head of Pediatric Intensive Care Unit, Maternity and Children Hospital, Buriadah – KSA; 2 Consultant, Pediatric critical care, Maternity and Children Hospital, Buriadah; 3 Resident, pediatric critical care, Maternity and Children Hospital, Buriadah; 4 Resident, pediatric department, Maternity and Children Hospital, Buriadah; 5 Consultant pediatric cardiologist, Prince Sultan cardiac center, Qassim; 6 King Abdullah International Medical Research Center/King Saud bin Abdulaziz University for Health Sciences, Department of Biostatistics and Bioinformatics-MNGHA, Riyadh, 11426, Saudi Arabia; 7 Assistant professor, College of Medicine, Alfaisal University Consultant, Pediatric Critical Care Section, Pediatric department King Faisal Specialist Hospital & Research Center, Riyadh; 8 Consultant, Pediatric critical care, Children Hospital, King Saud Medical City, Riyadh KSA

## Abstract

**Objective:**

To examine the accuracy of our national Life-Saving Protocol (LSP). To the best of our knowledge, this is the first study addressing this issue in Saudi Arabia.

**Background:**

LSP was created to facilitate triaging patients with LIFE or LIMB threatening conditions in peripheral hospitals with limited services to large regional hospitals to receive definitive care.

**Method:**

This is a retrospective single-center observational study over 12 months studying the patients who arrived via LSP to our Emergency room (ED), at the only regional pediatric hospital. For the subgroup of patients who were admitted to PICU through LSP, we further assessed their outcomes like mortality and length of stay (LOS) through a matched case-control study of 1:1 with similar patients who were admitted to our PICU via other routes rather than LSP. The primary outcome is to assess the accuracy of the LSP in triaging pediatric patients with LIFE of LIMB conditions. Secondary outcomes include assessing the association between LSP and (mortality, LOS) for those who were admitted to the regional PICU via LSP compared to patients admitted to PICU via other sources of admission.

**Results:**

During the study period, 118 patients arrived at our ED via LSP. Only 43 patients (36 %) were admitted to the PICU with LIFE or LIMB conditions. A total of 64 patients (54%) of the patients were admitted directly to the general pediatric ward from ED level due to absence of LIFE of LIMB threatening condition and 8% (n=9) were discharged immediately home from the ED level due to lack of any significant illness. One patient died at ED level, and one was referred to another hospital with a minor orthopedic injury. For those who were admitted to the PICU via LSP, the mortality rate was (13.9%) (6/43), and the control group was (4.6%) (2/43) with a p-value of 0.08.

**Conclusion:**

LSP is an excellent initiative and essential tool in our healthcare system; however, our study showed huge variation in the ability of the system to recognize true pediatric patients with LIFE or LIMB conditions. Our study might form a stepping-stone in future studies assessing the LSP at the national level.

## Introduction

Many studies have demonstrated improved survival for critically ill pediatric patients cared for in dedicated regional pediatric critical care units (PICU) compared with small or nontertiary PICUS. [1–3] There is considerable variation in outcome of the transferred patients due to the variation in the composition of the transport team and the duration of the transport. [4] The transfer process itself is associated with high resource utilization and high odds of mortality in both adult and pediatric. [4,5] The importance of these findings is being reflected in the recommendation of international organizations such as the Society of Critical Care Medicine (SCCM) and the American Academy of Pediatrics (AAP) to advocate for the regionalization of pediatric critical care services in the United States. [3,6] Given the overall limited availability of PICU space and staff, it’s imperative that only patients who absolutely require this service be referred to the regional PICUs; therefore, there is abundant literature and excellent reports investigating the efficiency and accuracy of triaging and transferring critically ill pediatric patients from remote (community) hospital to regional PICUs [7,8].

It’s well recognized that critically ill patients might get worse overtime whenever specialized ICU care is lacking; hence, the inter-hospital transfer is necessary (transferring of critically ill patients between hospitals). The inter-hospital transfer may save lives, but it is expensive, logistically challenging, and not without risk. The patient’s transport process by itself carries a risk for clinical deterioration and adverse events. These adverse events are proportional to the duration of the transfer, the pre-transfer severity of illness, and the experience of the medical transport team. [9] The key to the successful inter-hospitals transfer of critically ill patients is stabilization before transport. [10] The decision to transfer a patient to another hospital must be made by the most responsible physician in collaboration with other colleagues from relevant specialties and subspecialties in both referring and receiving hospitals [11].

**Figure 1:**
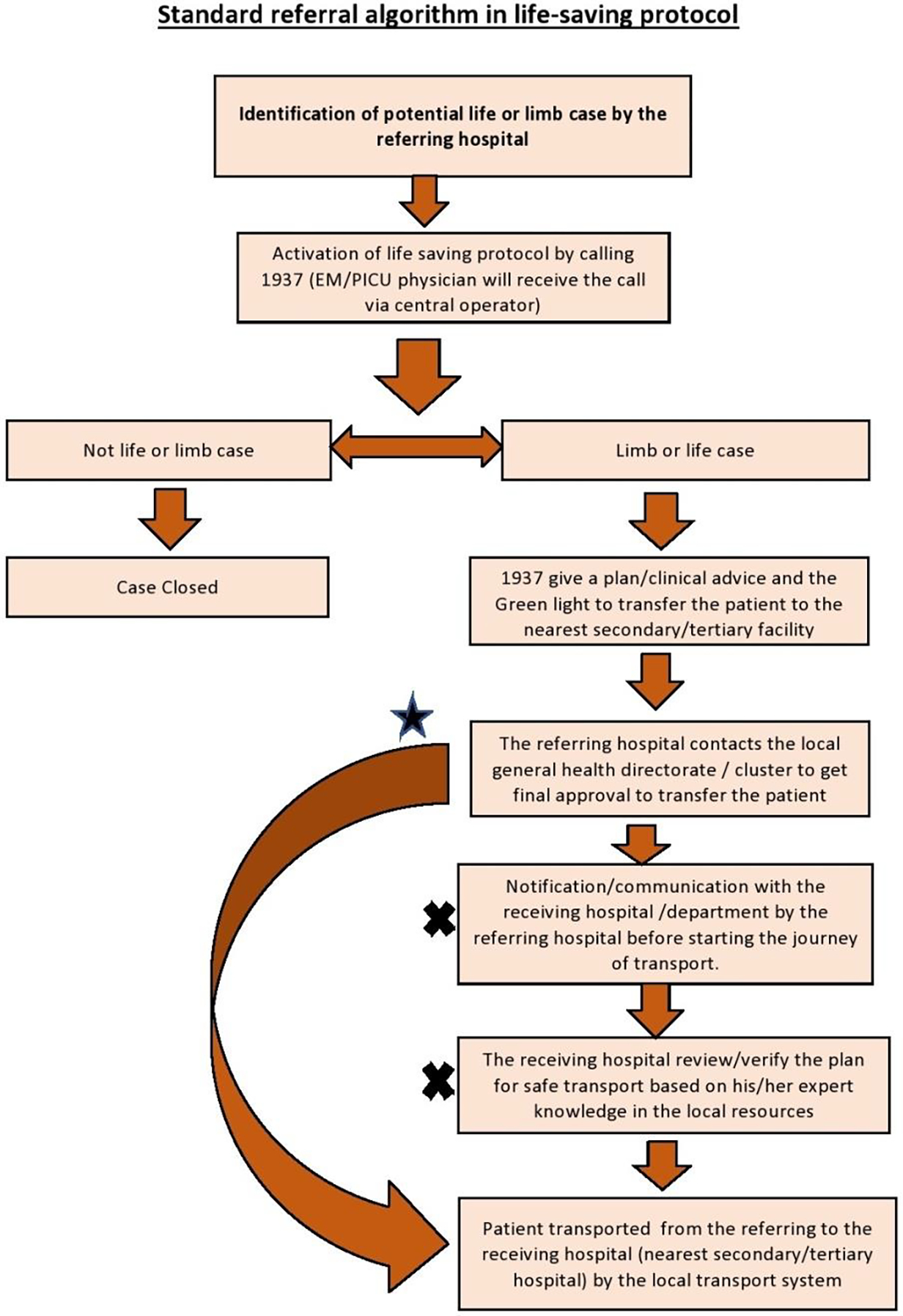
Referral flow (*) The star and the long arrow represent an unapproved alternative route that is commonly seen in the referring cases (deviation from the standard). (X) The x represents commonly overlooked steps in the process. (deviation from the standard).

The Kingdom of Saudi Arabia is a vast country with 13 regions (provinces) distributed over approximately 2,150,000 km^2^. [12] This large surface area, coupled with the global shortage of critical care services, led to the creation of life-saving protocol as the national triaging system under the Ministry of Health (MOH) in Saudi Arabia. The life-saving protocol is a national health care system that allows remote peripheral hospitals to access specialized care through one-number to call and transfer their critically ill patients to secondary or tertiary hospitals once the patient needs a medical/surgical service that is not available at the referring hospital. The system’s motto has always been (the patient first and the bed second), which is a great motto where the efforts are channeled to provide the best care for the critically ill patient without getting caught up in the logistics of bed availability and the receiving hospital approval. However, transferring a critically ill patient to the designated large regional hospital without its pre-transfer communication, approval, and arrangements could create unintentional problems. (Figure 1) (see supplements 1,2 for more details regarding the Life-saving protocol in MOH).

As with any system, life-saving protocol varies across the regions, given the variations among the region’s characteristics that lead to variations in the service and, eventually variations in the system’s outcome. There is no one-size-fits-all when it comes to a health care system that provides a special service for a 30 million population in a large country like Saudi Arabia. We clearly understand the inherited limitation and the need to optimize each region’s system to match the region-specific characteristics in terms of supply and demand.

In the Qassim region, there is a centralized command center located in the general directorate for the health system (replaced now by a command center in the Qassim cluster). The usual process in handling the calls at the command center used to be as the following; the center usually receives all transport requests for both adult and pediatric patients from remote peripheral hospitals. These requests are typically received by phone calls as well as fax with a special form to be filled by the referring hospital (supplement 1). Approved patients’ transfer requests will be sorted accordingly, and then patients will be transported to the appropriate designated secondary/tertiary hospital. However, this process lacks a clear communication/consultation with the experts specialized critical care where the one triaging the calls at either the general directorate for health system or Qassim cluster center is not qualified by certificate and training in the pertained subspeciality in each individual call. Recently, this problem had overcome by the unified national number (1937), which was introduced into the system as a part of the life-saving protocol in which a qualified physician by certificate and training depends on each individual call (i.e. NICU, PICU, adult ICU patients), is receiving the calls through call-conference to approve if this patient is considered as LIFE or LIMB case. He or she also offers medical advice over the phone to the treating team prior to the transfer to the designated hospital if deemed necessary; moreover, this service is available 24/7.

It’s well known that any inter-hospital transport system will improve patient outcomes with a prerequisite of the pediatric-specific system and logistics; however, we don’t have a dedicated pediatric transport team and most peripheral hospitals use their own transport resources to transfer the patients to the designated regional hospital [13].

Finally, Given that our hospital is the only tertiary pediatric hospital in the Qassim region, we attempted in our study to (a) evaluate the efficiency and efficacy of the life-saving protocol in its current form (b) to assess the outcome of pediatric patients admitted to our PICU through this system by looking at mortality and length of stay knowing that outcome of PICU patients may vary according to the source of admission to PICU (admitted to PICU from pediatric ward, ED, OR or from other small PICU) [14].

## Methods and Materials

### Study settings

Qassim Region is located almost at the center of the kingdom of Saudi Arabia. Qassim population is around 1, 6 million, and 24% of this population is less than 14 years of age, representing the cut off age for pediatric patients in our country. [15] There are 19 hospitals (including secondary and tertiary) and 181 primary health care centers that provide urgent and non-urgent medical services to citizens and visitors to the region [15].

Maternity and children hospital (MCH) is located in Buriadah -the capital city of the Qassim Region and it is the only pediatric standing hospital in the region. MCH was established in 1982 and has become one of the most important hospitals in Buriadah City with a bed capacity of 500 beds. The hospital provides specialized medical/surgical care in Pediatrics and Obstetrics for Buriadah City and all Qassim Region with an accredited training program in pediatrics. Our pediatric intensive care unit (PICU) is a seven-bed closed system PICU dedicated to critically ill children from 0–14 years old. The average total admission per year is around 500 patients with a mean length of stay of 4 days and an occupancy rate of 85%.

In general, critically ill children arrive at our hospital ED through different pathways:

- Regular transport from other hospitals after direct acceptance through fax/Ehality system by MCH staff,
- Life-saving protocol utilizing peripheral hospital transport but most of the time without prior acceptance or approval from MCH staff.
- Through the red crescent transport system especially for trauma patients on the road; however, sometimes the family bring their own critically ill child directly to ED. Our aim is only to study patients arriving through a life-saving protocol.

### Study design / Method

The study is a retrospective single-center observational study carried out over 12 months period (from January 2018 to December 2018). For the subgroup of patients who were admitted to PICU through life-saving protocol, we further assessed their outcomes like mortality and length of stay (LOS) through a matched case-control study of 1:1 with similar patients who admitted to our PICU via other routes rather than life-saving protocol where we defined the cases as patients admitted to our PICU via the life-saving protocol and the control matched age and diagnosis as patients who admitted to our PICU directly from our ED or pediatric ward without being transferred via the life-saving protocol. The inclusion criteria were all pediatric patients aged 0–14 years old who came to ED via the life-saving protocol. Exclusion criteria were any pediatric patients who came to ED through other systems like; fax acceptance, ED drop-in by family, Red Crescent, or patients transferred between pediatric intensive care units with prior acceptance.

**Figure 2:**
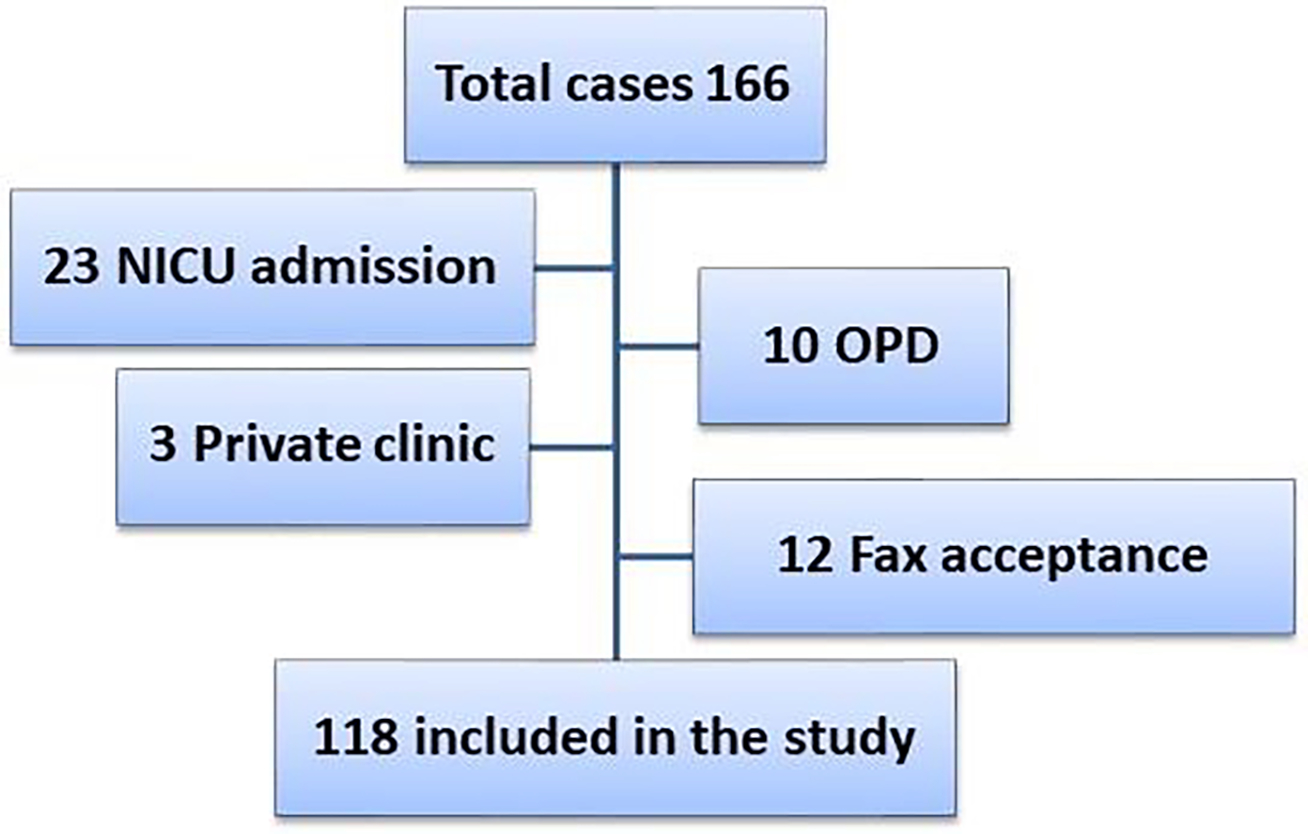
Flow chart for patients screening for eligibility.

We screened the hospital records (the electronic and the paper records) for all eligible patients according to inclusion & exclusion criteria (see flow chart: Figure 2). We used a case report form (CRF) for data collection, which captured demographic data of patients, the reason for referral, other data relevant to the outcome, e.g. (Pre transport complication, transport complication, co-morbidity, assessment at our emergency department, length of stay (LOS). Then these CRFs are converted to the excel spreadsheet to be finally analyzed by SPSS software.

All patients’ identifiers were removed for patient privacy and confidentiality, and ethical approval was secured from the regional ethical committee before starting the study.

### Statistical analysis of the data

Data were analyzed using IBM^®^ SPSS^®^ Statistics V25. All continuous data are expressed as mean ± standard deviation unless otherwise specified. Continuous, numerical data were compared between groups using the student t-test. Discrete / categorical variables were compared between the two groups using the χ 2 test. Univariate analysis of risk factors for mortality was performed using the Cox proportional hazards test. The threshold for statistical significance was p < 0.05.

## Results

During the study period, total cases screened were 166; however, only 118 were included after applying the exclusion criteria (Figure 2).

**Figure 3:**
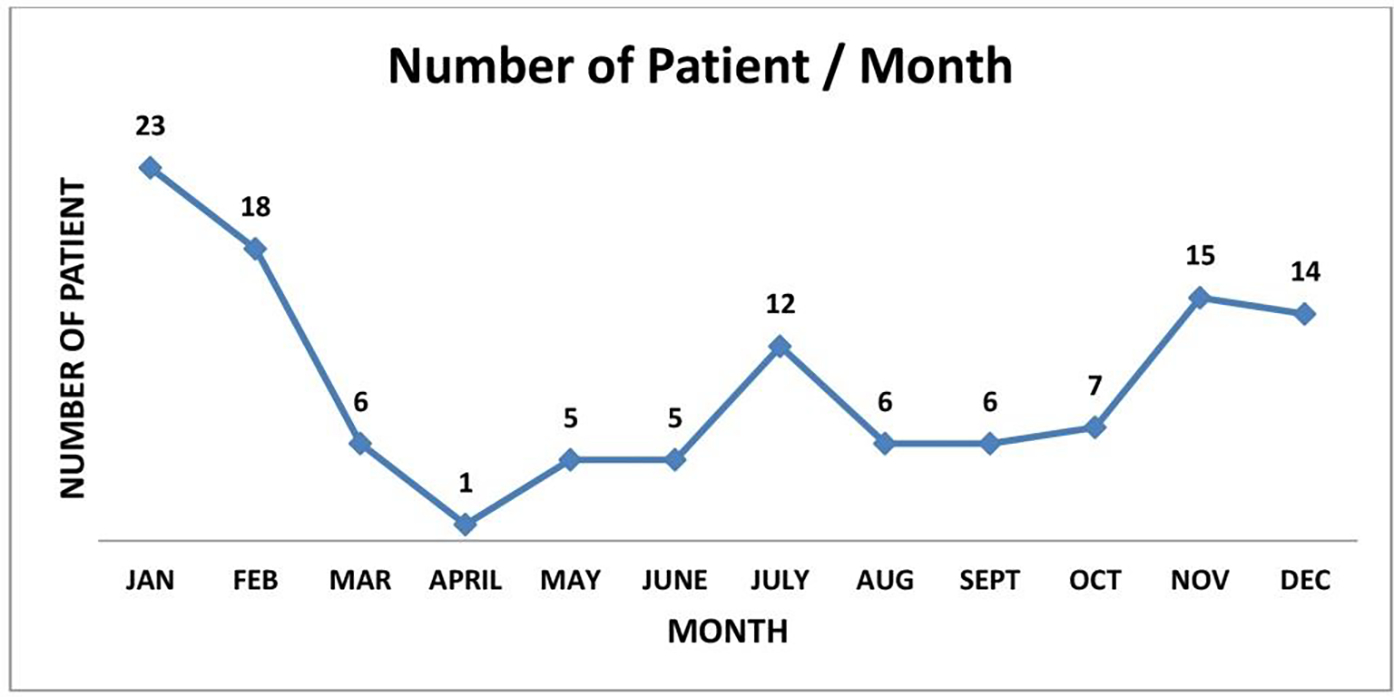
Seasonal distribution of patients arrived via life-saving protocol.

A total of 118 patients arrived at our ED in MCH via the life-saving protocol; 58% (n=68) were males, and the remaining %42 (n=50), were females with a mean age of 58.61 months (Table 1). The major part of transfer events happens during the winter season (over 4 months, November- February) (Figure 3).

Quality of communication between hospitals cannot be assessed rigorously due to missing data, where we found 76% of the referred cases have no documented notes regarding the nature of communication between the two hospitals.

The majority of transfers stratified per city were transferred from Buriadah City (31.3%) (n=37), given that it’s the capital of Qassim region as well as the largest hospitals in Buridah city (BCH and KFSH) lack pediatric services and usually transfer all their pediatric patients to us in MCH (Supplement 3).

The main reason for referring patients to our pediatric intensive care unit (PICU) was CNS illnesses ( 29 %) (n=34) followed by reparatory illness (27%), (n=32). The co-morbidity were present in 33.1% (n=39) of all the patients (defined as patient known to have at least one chronic disease before the transfer event). Total of 19 patients, (16.9%) of the transferred patients were ventilated, and only two patients were on inotropic support. Cardiorespiratory Resuscitation (CPR) was performed in 5.2% (n=6) patients before the transfer (Table 1).

**Figure 4:**
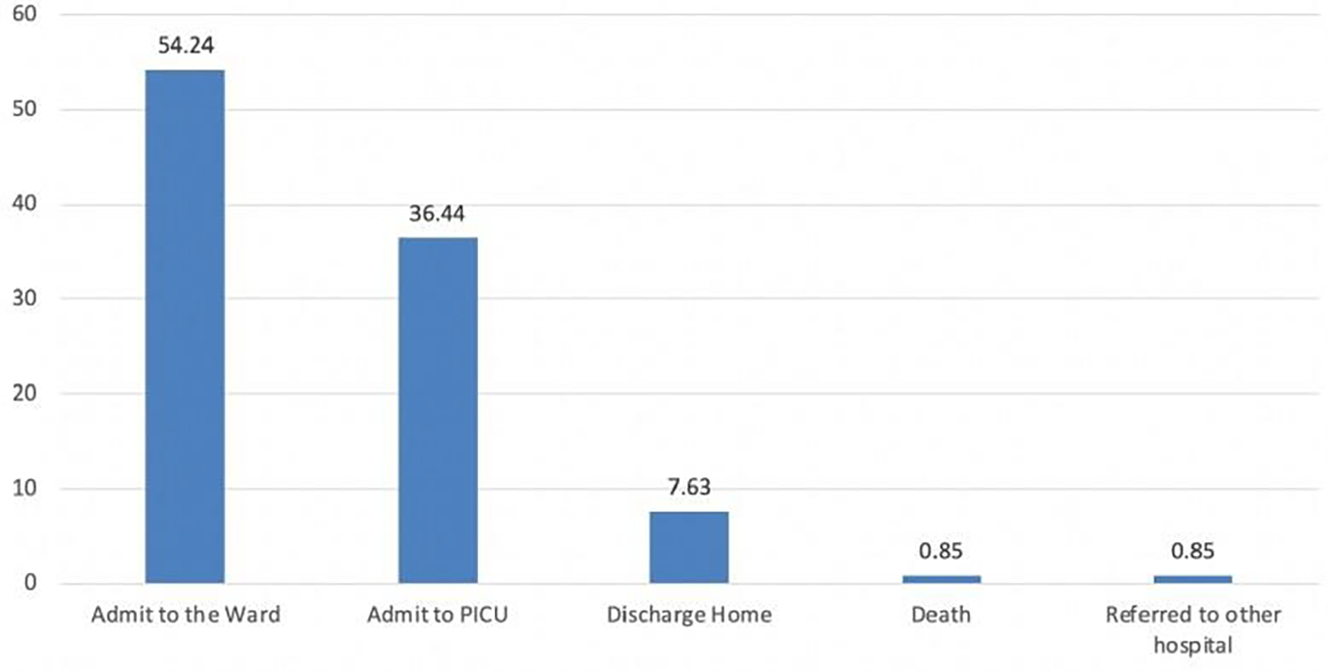
Disposition of all patients arrived via the protocol at ED level.

Total of 64 patients (54.2%) of the transferred patients via life-saving protocol were admitted to the general pediatric ward instead of PICU, which is the original intention of the system (to transfer only patients with LIFE or LIMB conditions to the regional critical care unit). Only 36.4% (n=43) were admitted to PICU, and surprisingly 7.6% (n=9) were discharged home from ED level after being deemed to have a mild illness that did not even need admission to the pediatric ward. One patient died at ED, and one was referred to another hospital with a minor orthopedic injury (RT metatarsal bone fracture, given there is no orthopedic service in MCH (Figure 4).

In the subgroup analysis of the 43 patients who were admitted to PICU via the life-saving protocol, we found them to be young with a mean age of 58 months and 55.8% (n=24) male. Almost half of them (n=19 patients) were ventilated, and only two were on inotropic support.

We did a match analysis for those 43 patients and compared the outcomes of LOS and PICU mortality (Table 2). There were no differences in the mean age and PICU length of stay between cases and controls. The rate of mortality was more in the case-cohort (13.9%) (n=6/43) as compared to 4.6% (n=2/43) in the control cohort; however, there was no statistical significance with a p-Value of 0.08. Of note, the six patients who died in the cases (the referred) were as follows: one encephalitis with brain edema, one chest infection and ARDS, one severe TBI, one out-hospital cardiac arrest, one with sepsis catecholamine-refractory, and one with AV malformation. The causes of death in the control group were as follow: one patient with sickle cell disease with ARDS and the second was a Cerebral palsy (CP) kid with global developmental delay (GDD) who came with status epilepticus and respiratory failure. Unfortunately, no severity scores were established in either the referral or the receiving hospital, which might be a valuable tool to assess the system and our PICU.

Multivariable logistic regression was used to determine the relationship between variables like (age, time of admission) and specific outcomes (mortality, PICU admission) however, no significant association was found (see supplements 4, 5 for multivariable logistic regression).

## Discussion

In general, Inter-hospital transfer to a specialized regional facility is a complex process. Its success depends on the shared mental model between the leaders (admin/managers in the health care system) and the end-users (the health care providers at the front lines). We have many successful adult medical care initiatives, like the stroke and STEMI programs, where a specialized health professional manages a subgroup of very sick patients within a short period (identified window). Another good international example of this integrated system is the life or limb policy in Ontario province in Canada, where the ministry of health in Canada collaborated with local critical care services to establish a system where the patient’s life or limb-threatening condition is a priority, and the identification of beds is a secondary consideration [16].

The shared mental model between the leaders and end-users is translated into critical components (like the chain of survival in advanced life supports). Any break in any component will have a downstream effect on the quality of care and patient outcome. The components of an effective life-saving system in critical care could be broken down into five guiding principles:

1. Criteria/guidelines for early identification of targeted patients (screening system) with reasonable sensitivity to pick up only critically ill patients,
2. Communication system to facilitate direct communication between the referral team who trigger/activate the system and with the end-user (most qualified health care provider who will provide definite care in the receiving hospital),
3. Transport system to guarantee safe transport of the critically ill patients to the designated facility,
4. Repatriation protocol to the original hospital after definite management have been provided, and
5. Auditing system with ongoing monitoring for the system components through a set of agreed-upon indicators [16, 17] (Table 3).

It is vital to have a strategy at the Macrosystem level (regional level or even at the national level) with an organized health care system dedicated to critical care management to ensure that every critically ill patient receives care of the highest quality and the lowest cost. However, such a system should be calibrated based on the supply and demand at the regional level (a load of patients, number of experts/centers, the geographical distribution of population, and distance to the specialized facility) to match the need of the best cost-effective way possible [15,16,17].

Examining the integrated transfer system components for the inter-hospital transfer (or life-saving protocol) and performing a deliberate, reflective analysis of our study’s findings, we identified many areas of improvement in our region.

For the first component (1) early identification of critically ill pediatric patients who need regional PICU services (screening system), the goal is no critically ill patient will be refused to care for. The priority is for the required service in the regional facility regardless of beds’ availability in the receiving facility. However, it looks like the system in our area lacks a pediatric-specific triaging system with low sensitivity in the pediatric population, where we found only about one-third of those who came via the life-saving protocol were actually admitted to PICU. In comparison, approximately 55% were admitted to the general pediatric ward, and surprisingly, about 8% were discharged home from ED level in the receiving hospital (which is considered a waste in quality terms).

For the second and third components of integrated inter hospital transfer system (2) communication system and (3) transport system. Although the referral facility communicated with the local authority to approve the transport as well as communicated with medical staff over the national call number, however, we could not assess the quality of communication beyond this level due to missing data (76%), with only 15% of these transfer events were clearly documented the events of communication with the receiving hospital before the actual transport. This factor is often overlooked during the rush of transferring a critically ill patient. However, it is imperative that the patient received the basic resuscitation and stabilized before the transport (there were some patients with septic shock with no IV line, and no antibiotics were given before transfer, only given at our ED level). Pre-transfer intervention is linked directly to the outcome of critically ill patients, and communication with the receiving pediatric intensivist prior to the transfer is the best way to ensure optimal pre-transfer condition [16, 17].

The fourth component (4) repatriation to the original hospital after definite management is of paramount importance in the context of one regional PICU with limited capacity and space serving a large population distributed over a vast geographical area. Without a robust regional- repatriation system, the regional PICU beds will rapidly be occupied, and staff will be overwhelmed with less critical (or stable recovering critically ill patients) with no way to serve the newly-referred critically ill patients who are waiting for beds in ED [15, 16]. Unfortunately, no repatriation system existed in the region and all patients who came to our tertiary hospital stay until discharged home or repatriated with personal efforts to their original community hospital.

The fifth (5) and last component: an auditing system with ongoing monitoring for the system components through a set of agreed-upon indicators is a vital tool to assess and recalibrate the system periodically based on the real input from the real world; hence it’s a dynamic process. Indicators should reflect the critical part of the system as well as the clinical outcomes. Unfortunately, we have only a few administrative indicators with no clinical indicators like utilization of PICU-specific intervention, LOS, and mortality. In addition, there is no current method to evaluate the accuracy of the triaging system as well as no clear plan to assess the transport system given that all triaged and transported cases from both adult and pediatric are lumped under one indicator with little clinical value.

## Limitation

Our study has major limitations in form of being a single-center as well as with many missing data that preclude sound and scientific conclusions. In addition to that, the low number of patients in the PICU subgroup and lack of formal score of severity, statistically weaken any conclusion regarding the outcomes. Another limitation is the data were collected on 2018 and there might be new updates to the life-saving protocol and this dataset doesn’t reflect the current status of the system.

## Conclusion

Life-Saving Protocol is an excellent initiative and important tool in our healthcare system; however, our study showed huge variation in the ability of the system to recognize true pediatric patients with LIFE or LIMB conditions. Although our study is unique to our region, it might form a stepping-stone in the future studies in assessing the system at the national level in different regions and identify the gaps and the areas for improvements

## Supplementary Material

1

## Figures and Tables

**Table 1:** Demographic data and characteristics for all patients who arrived at ED via life-saving protocol (n=118).

Characteristic	Group	Percentage (number)
Gender	Males	58% (68)
	Females	42% (50)
Age Group	3 months or less	13% (15)
	>3 to 6 months	4% (5)
	>6 to 12 months	10% (12)
	> 12 months to 24 months	12% (14)
	> 24 months	61% (72)
Time of referral	> 8:00 to 16:00	29% (34)
	> 16:00 to 00:00	41% (49)
	00:00 to 8:00 AM	30% (35)
IV line access	Absent	24% (28)
	Present	62% (73)
	Not recorded	14% (17)
Ventilation	Not ventilated	78% (92)
	Ventilated	16% (19)
	Not recorded	6% (7)
History of CPR	No CPR	89% (105)
	Yes	5% (6)
	Not recorded	6% (7)
Communication before transfer	Not clear	76% (90)
	NO	8% (10)
	YES	15% (18)
Organ System Involved	CNS	29% (34)
	Respiratory	27% (32)
	Endocrine	7% (8)
	Multiorgan	9% (11)
	Hematology	8% (10)
	GIT	14% (16)
	RTA	4% (4)
	Surgical	3% (3)
	Total	118

**Table 2:** Comparison between the referred life–saving cases and matched control patients from our PICU (matched for diagnosis, age, gender).

Variable	Cases (total of 43 patients)	Control (total of 43 patients)	P-value
Age (Mean & SD)	57.12 (58.3)	53.91 (52.6)	0.783
PICU LOS (Mean &SD)	5.56 (6.3)	4.0 (8.6)	0.351
Males (No & %)	24 (56%)	27(63%)	0.33
Females (No &%)	19 (44%)	16 (37%)	
Alive (No & %)	37 (86%)	41 (95.4%)	0.08
Dead (No & %)	6 (13.9%)	2 (4.6%)	

**Table 3:** Components of an effective life-saving system in critical care.

Name of the component	Description / benefits
screening system	Clear guidelines/criteria for early identification of targeted patients with reasonable sensitivity for picking up only critically ill patients
communication system	The standard method that facilitates easy and effective communication between the referring team and the receiving team once potential patients are identified
Transport system	Standard transport system with designated retrieving team, who are experts in the targeted population by certificate.
Repatriation protocol	Policy for repatriation after 48 of stability and /or definitive care has been accomplished to keep open access in secondary/tertiary hospital for potential future patients.
Auditing system	A centralized system that is able to record/retrieve calls and documents and supervised by an expert to generate feedback/recommendation/corrective action/training/policy and procedure revision as needed.
