# Supplementary material for: Assessing Pediatric Inter-Hospital Transfer: A single-center, Retrospective, Observational Study of Saudi Arabia’s National Life-Saving Protocol
Source: J Pediatr Perinatol Child Health. Author manuscript; Available in PMC 2023 Jan 26. (PMC9879325; doi:10.26502/jppch.74050130)
Supplement: 1 [file NIHMS1863212-supplement-1.pdf]

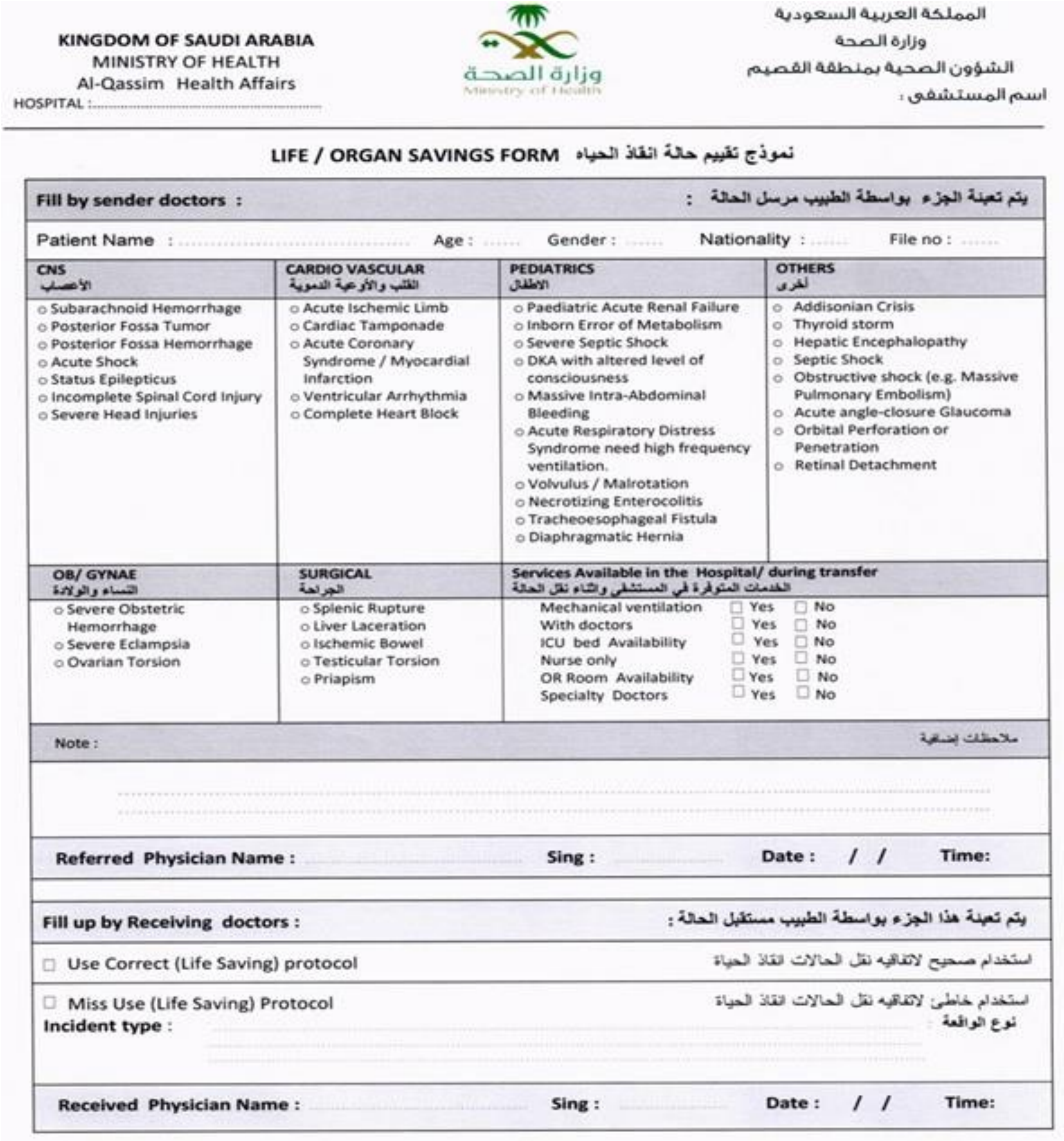

KINGDOM OF SAUDI ARABIA
MINISTRY OF HEALTH
Al-Qassim Health Affairs
HOSPITAL :..............................

وزارة الصحة
Ministry of Health

المملكة العربية السعودية
وزارة الصحة
الشؤون الصحية بمنطقة القصيم
اسم المستشفى :

LIFE / ORGAN SAVINGS FORM نموذج تقييم حالة انقاذ الحياه

Fill by sender doctors : يتم تعبئة الجزء بواسطة الطبيب مرسل الحالة :

Patient Name : .... Age : ...... Gender : ...... Nationality : ...... File no : ......

CNS الأعصاب	CARDIO VASCULAR القلب والأوعية الدموية	PEDIATRICS الاطفال	OTHERS أخرى
○ Subarachnoid Hemorrhage<br>○ Posterior Fossa Tumor<br>○ Posterior Fossa Hemorrhage<br>○ Acute Shock<br>○ Status Epilepticus<br>○ Incomplete Spinal Cord Injury<br>○ Severe Head Injuries	○ Acute Ischemic Limb<br>○ Cardiac Tamponade<br>○ Acute Coronary Syndrome / Myocardial Infarction<br>○ Ventricular Arrhythmia<br>○ Complete Heart Block	○ Paediatric Acute Renal Failure<br>○ Inborn Error of Metabolism<br>○ Severe Septic Shock<br>○ DKA with altered level of consciousness<br>○ Massive Intra-Abdominal Bleeding<br>○ Acute Respiratory Distress Syndrome need high frequency ventilation.<br>○ Volvulus / Malrotation<br>○ Necrotizing Enterocolitis<br>○ Tracheoesophageal Fistula<br>○ Diaphragmatic Hernia	○ Addisonian Crisis<br>○ Thyroid storm<br>○ Hepatic Encephalopathy<br>○ Septic Shock<br>○ Obstructive shock (e.g. Massive Pulmonary Embolism)<br>○ Acute angle-closure Glaucoma<br>○ Orbital Perforation or Penetration<br>○ Retinal Detachment

OB/ GYNAE النساء والولادة	SURGICAL الجراحة	Services Available in the Hospital/ during transfer الخدمات المتوفرة في المستشفى وأثناء نقل الحالة		
○ Severe Obstetric Hemorrhage	○ Splenic Rupture	Mechanical ventilation	☐ Yes	☐ No
○ Severe Eclampsia	○ Liver Laceration	With doctors	☐ Yes	☐ No
○ Ovarian Torsion	○ Ischemic Bowel	ICU bed Availability	☐ Yes	☐ No
	○ Testicular Torsion	Nurse only	☐ Yes	☐ No
	○ Priapism	OR Room Availability	☐ Yes	☐ No
		Specialty Doctors	☐ Yes	☐ No

Note : ملاحظات إضافية

....
....

Referred Physician Name : .... **Sing :** .... **Date :** / / **Time:**

Fill up by Receiving doctors : يتم تعبئة هذا الجزء بواسطة الطبيب مستقبل الحالة :

☐ Use Correct (Life Saving) protocol استخدام صحيح لاتفاقيه نقل الحالات انقاذ الحياة

☐ Miss Use (Life Saving) Protocol استخدام خاطئ لاتفاقيه نقل الحالات انقاذ الحياة

Incident type : .... : نوع الواقعة
....

Received Physician Name : .... **Sing :** .... **Date :** / / **Time:**

Supplement 1: Life-saving form.

التجمع الصحي بالقصيم
Qassim Health Cluster

عنوان السياسة	سياسة إحالة مرضى حالات إنقاذ حياة/عضو بين المستويات الصحية			
جهة الإصدار		رقم السياسة	يستبدل السياسة رقم	مستوى السياسة
إدارة التنسيق للإحالات الطبية		C3-EHT-PP-002-A-V1	جديد	التجمع الصحي
تاريخ الاعتماد	تاريخ التطبيق	تاريخ المراجعة	عدد الصفحات	تطبق على
15-03-2020	01-04-2020	10-03-2020	2	أقسام التنسيق للإحالات الطبية بالمستشفيات

١. الغرض:

1.1: تحديد سياسات وإجراءات تنظيم عمل إحالة مرضى إنقاذ حياة/عضو بين المستويات الصحية داخل وخارج منطقة القصيم.

٢. التعريفات:

2.1: حالات إنقاذ الحياة/عضو : الحالات المهددة لحياة المريض أو عضو من أعضائه وتحتاج لخدمة طبية عاجلة أو تدخل طبي أو جراحي معين لا يمكن توفيره بنفس المستشفى المتواجد به المريض.

٣. السياسات:

3.1: سياسة العمل للحالات إنقاذ حياة/عضو بين المستشفيات.

الإجراءات :

4.1: اجراءات الطبيب المحيل :

4.1.1: إذا قرر الطبيب المعالج نقل حاله انقاذ حياة/عضو إلى أي مستشفى أخر تتوفر فيه الخدمة فانه يقوم بالاتصال على مركز ١٩٣٧ لأخذ الموافقة من عدمها وتدوين اسم الطبيب المناوب في المركز.

4.1.2: في حاله موافقة مركز ١٩٣٧ يقوم الطبيب المعالج بإعداد التقرير الطبي بالنموذج الموحد للحالة أنقاذ حياه/عضو موضحا سبب الإحالة وتسليمه إلى قسم التنسيق للإحالات الطبية بالمستشفى .

4.1.3: يرفق الطبيب مع التقرير كل المرفقات المطلوبة. مثل (نموذج انقاذ الحياة المعتمد من قبل وزارة الصحة– معلومات المريض – صورة الهوية – نتائج الأشعة والتحاليل أن وجد).

اجراءات المستشفى المحيل:

4.1.1.1: يقوم المنسق الطبي فور استلام المستندات بالتأكد من صحتها واكتمالها وعمل مسح ضوئي بصيغة pdf.

4.1.1.2: الاتصال بعمليات التنسيق الطبي بالتجمع الصحي لإخذ توجيه من الطبيب المناوب بالمستشفى الذي يستقبل الحالة.

4.1.1.3: اختيار نوع الحالة (أنقاذ حياه/عضو) في (نظام التحويل الالكتروني – إحالتي) وتعبئة البيانات المطلوبة مع ضرورة كتابة اسم الطبيب المناوب في مركز ١٩٣٧ الذي سمح بنقل الحالة في خانة السبب التفصيلي للإحالة.

4.1.1.4: اختيار التخصص المطلوب والتخصص الدقيق بدقه حسب التقرير الطبي وإرفاق المستندات التي تم مسحها بصيغه pdf.

4.1.1.5: يقوم المنسق الطبي بتأكيد النقل عبر برنامج احالتي والأشراف على نقل الحالة بالتنسيق بين النقل الإسعافي وإدارة التمريض والطبيب المعالج وحل المعوقات إلى إن تصل إلى المستشفى المستقبل.

: اجراءات المستشفى المستقبل:

4.1.2.1: يقوم المنسق الطبي بالمستشفى المستقبل فور استلام الطلب بطباعة المستندات المرفقة وتسليمها لقسم الطوارئ والطبيب المختص .

4.1.2.2: يلتزم قسم الطوارئ بإشعار قسم التنسيق للإحالات الطبية بوصول المريض ويتم تأكيد الوصول عبر برنامج احالتي.

C3-EHT-PP-002-A-V1Page 1of 2

عنوان السياسة	سياسة إحالة مرضى حالات إنقاذ حياة/عضو بين المستويات الصحية			
جهة الإصدار		رقم السياسة	يستبدل السياسة رقم	مستوى السياسة
إدارة التنسيق للإحالات الطبية		C3-EHT-PP-002-A-V1	جديد	التجمع الصحي
تاريخ الاعتماد	تاريخ التطبيق	تاريخ المراجعة	عدد الصفحات	تطبق على
15-03-2020	01-04-2020	10-03-2020	2	أقسام التنسيق للإحالات الطبية بالمستشفيات

4.1.3.3: يقوم المنسق الطبي بالإشراف على وصول الحالة وحل أي معوقات تواجه المريض أو الطاقم المرافق وإنهاء جميع إجراءات الطاقم المرافق (توقيع الانتدابيات وختمها).

4.1.3.4 : في حال اتضح للطبيب الذي استقبل الحالة أنها ليست إنقاذ حياة/عضو أو أن حالة المريض غير متطابقة تماما مع التقرير الطبي يتم تعبئة نموذج التغذية الراجعة (feed back) وإرفاقه بالمرفقات الإضافية للإحالة وإبلاغ إدارة التنسيق بالتجمع الصحي بذلك .

4.1.3.4: يلتزم قسم التنسيق للإحالات الطبية بالمستشفى المستقبل الإبلاغ عن حالة المريض وماذا تم بخصوصه بعد مضى ٢٤ ساعة وذلك بالمرفقات الإضافية للإحالة.

٤. المرفقات :

١- التقرير الطبي على أن يكون في النموذج الموحد المعتمد من الوزارة.
٢- الهوية الوطنية.
٣- استمارة معلومات المريض.

٥. المراجع :

١- دليل سياسات وإجراءات العمل بإدارة أهلية العلاج والتنسيق الطبي بصحة القصيم تاريخ ١٦-٠٥-٢٠١٨م.
٢- لجنة مراجعة سياسات وإجراءات العمل بصحة القصيم تاريخ / ١٢-٠٩-٢٠١٩م ورقم / ٢٧٠٤٣٨.
٣- التجارب الإقليمية و العالمية : بريطانيا ، ، السويد سويسرا ، هولندا ، منظمة الصحة العالمية.
٤- تجربه مستشفى الملك فيصل التخصصي ومركز الأبحاث بالرياض.
٥- دليل نظام الإحالة بين المرافق الصحية : الإدارة العامة للمستشفيات ٢٠٠٨م.

٦. الاعتماد

الإعداد:

الاسم	المسمى الوظيفي	التوقيع	تاريخ
شليويح عطاالله المطيري	رئيس قسم التنسيق الطبي		
د. فتح الرحمن بشارة	المشرف الفني بإدارة التنسيق		

المراجعة:

الاسم	المسمى الوظيفي	التوقيع	تاريخ
محمد رشيد الرويلي	مدير إدارة التنسيق للإحالات الطبية		
د.عبدالاله محمد الحديثي	نائب الرئيس التنفيذي لرعاية الصحية		

الاعتماد:

الاسم	المسمى الوظيفي	التوقيع	تاريخ
د. سلطان سعود الشايع	الرئيس التنفيذي		

C3-EHT-PP-002-A-V1Page 2of 2

Supplement 2: Llife-saving policy and procedure (in Arabic).

Hospital name	Frequency	Percent
BCH (Buriadah City)	25	21.2
BUKAREA HOSPITAL (Bukariah city)	23	19.5
ALRASS HOSPITAL (Alrass City)	16	13.6
KFSH (Buridah City)	11	9.3
ALASYAH HOSPITAL (Alasyah City)	7	5.9
KING SAUD HOSPITAL (UNAZIA City)	7	5.9
QIBA HOSPITAL (Qiba City)	6	5.1
QUSABIA HOSPITAL Qusabah city)	5	4.2
UYON ALJIWA HOSPITAL (Aljiwa City)	4	3.4
PHC	3	2.5
ALMOTHNAB HOSPITAL (Almothnab City)	3	2.5
ALQUARA HOSPITAL	3	2.5
KKH,HAIL (Hail City)	2	1.7
MCH HAIL (Hail City)	1	0.8
ALBDAYE HOSPITAL (Albdaye city)	1	0.8
ALNABHANEH HOSPITAL (Alnabhaneh city)	1	0.8
Total	118	100.0

Supplement 3: Distribution of patients arrived via the protocol as per referral hospital.

Patients Characteristics and group		ICU admission		Total	OR	95% CI	P value
		Yes	No				
Age Group	3 months or less	9	6	15	0.3750 to 3.7500	0.0333 to 14.6170	0.0953 (> 0.05)
	>3 to 6 months	4	1	5			
	>6 to 12 months	4	8	12			
	> 12 months to 24 months	4	10	14			
	> 24 months	22	50	72			
Gender	Males	24	44	68	0.89	0.4173 to 1.8978	0.456
	Females	19	31	50			
Time-of-referral	> 8:00 to 16:00	12	22	34	0.7969	0.3252 to 1.9528	0.6195
	> 16:00 to 00:00	17	32	49			
	00:00 to 8:00 AM	14	21	35			
Weekend Arrival/admission	Yes	12	20	32	1.0645	0.4595 to 2.4664	0.524
	No	31	55	86			
Ventilation	Not ventilated	34	58	92	0.806	0.2952 to 2.2005	0.421
	Ventilated	8	11	19			
	Not recorded	1	6	7			
CPR	No CPR	39	66	105	0.5909	0.1136 to 3.0724	0.5317
	Yes	3	3	6			
	Not recorded	1	6	7			
Involved System	CNS	13	21	34	1.3619 to 4.3953	0.4919 to 3.7709	0.5522 (>0.05)
	RESPIRATORY	10	22	32			

	GIT	3	13	16			
	MULTIORGAN	5	6	11			
	HEMATOLOGY	3	7	10			
	Endocrine	7	1	8			
	RTA	2	2	4			
	SURGICAL	0	3	3			
Alive/Dead	Alive	36	74	110	0.0695	0.0082 to 0.5865	0.0143
	Dead	7	1	8			
Total		43	75	118			

ICU admission Cross tabulation and referring hospital, age group, Time of referral, patient status, and organ system involved

Supplement 4: Multivariable logistic regression for PICU admission.

Patients Characteristics and group		Alive	Dead	Total	OR	95% CI	P value
Age Group	3 months or less	12	3	15	0.1429 to	0.0067 to 3.0627	0.249
	>3 to 6 months	5	0	5	0.2353		
	>6 to 12 months	12	0	12			
	> 12 months to 24 months	13	1	14			
	> 24 months	68	4	72			
Gender	Males	65	3	68	2.2569	0.5142 to 9.9066	0.2808
	Females	45	5	50			
Time-of-admission	> 8:00 to 16:00	31	3	34	0.9293	0.1470 to 5.8763	0.9379
	> 16:00 to 00:00	46	3	49			
	00:00 to 8:00 AM	33	2	35			
Weekend Arrival/admission	Yes	31	1	32	2.7468	0.3244 to 23.2558	0.3539
	No	79	7	86			
ventilation	Not ventilated	85	7	92	0.6746	0.0781 to 5.8271	0.7205
	Ventilated	18	1	19			
	Not recorded	7	0	7			
CPR	No CPR	97	8	105	0.8824	0.0457 to 17.0390	0.934
	Yes	6	0	6			
	Not recorded	7	0	7			
ICU admission	Yes	36	7	43	0.0695	0.0082 to 0.5865	0.0143
	No	74	1	75			
Involved System	CNS	31	3	34	0.2727	0.0133	0.3995
	RESPIRATORY	32	0	32	to	to	
	GIT	16	0	16	3.875	22.9575	
	MULTIORGAN	8	3	11			
	HEMATOLOGY	9	1	10			
	Endocrine	8	0	8			
	RTA	3	1	4			
	SURGICAL	3	0	3			

Alive/Dead	Yes	36	7	43	0.0695	0.0082 to 0.5865	0.0143
	No	74	1	75			
Total		110	8	118			

Mortality Cross tabulation and, age group, Time of referral, patient status, and organ system involved.

Supplement 5: Multivariable logistic regression for mortality.
